# Supplementary material for: Ritonavir Has Reproductive Toxicity Depending on Disrupting PI3K/PDK1/AKT Signaling Pathway
Source: Toxics. 2024 Jan 15;12(1):73. doi: 10.3390/toxics12010073 (PMC10819985; doi:10.3390/toxics12010073)
Supplement: Supplementary file 1 [file toxics-12-00073-s001.zip › toxics-2796831-supplementary.pdf]

Supplementary Material

Ritonavir has reproductive toxicity depending on disrupting PI3K/PDK1/AKT signaling pathway

Eun-Ju Jung[a], Jae-Hwan Jo[b], Claudine Uwamahoro[a], Seung-Ik Jang[a], Woo-Jin Lee[a], Ju-Mi Hwang[a], Jeong-Won Bae[a], and Woo-Sung Kwon[a,b,c,*]

[a]Department of Animal Science and Biotechnology, Kyungpook National University, Sangju, Gyeongsangbuk-do 37224, Republic of Korea

[b]Department of Animal Biotechnology, Kyungpook National University, Sangju, Gyeongsangbuk-do 37224, Republic of Korea

[c]Research Institute for Innovative Animal Science, Kyungpook National University, Sangju, Gyeongsangbuk-do 37224, Republic of Korea

*Corresponding author:

Department of Animal Science and Biotechnology, Department of Animal Biotechnology, and Research Institute for Innovative Animal Science

Kyungpook National University Sangju, Gyeongsangbuk-do 37224, Republic of Korea

Tel: +82.54.530.1942

Fax: +82.504.242.9851

E-mail: wskwon@knu.ac.kr

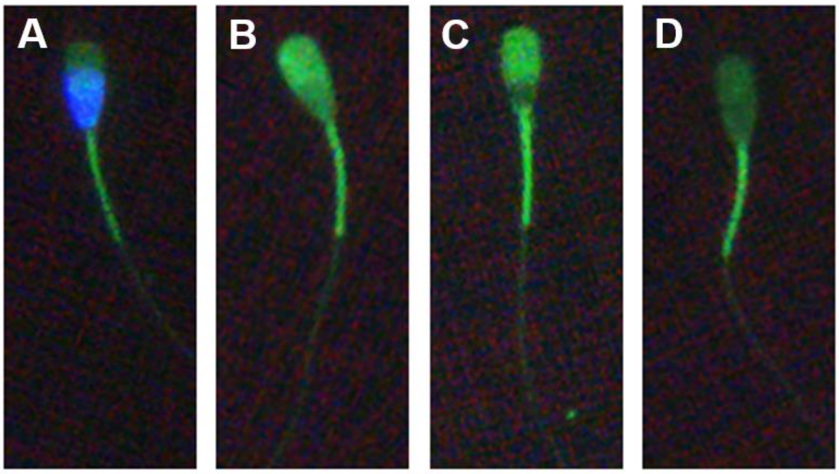

Supplementary Figure S1. Representative images of CTC patterns. (A) Dead sperm (D pattern, blue fluorescence on the sperm head). (B) Live non-capacitated sperm (F pattern, bright green fluorescence spread evenly on the sperm head). (C) Live capacitated sperm (B pattern, bright green fluorescence in acrosomal region). (D) Acrosome-reacted sperm (AR pattern, no fluorescence on the sperm head or bright green fluorescence only in the post-acrosomal region).
